# Hyponatraemia, AKI, and urinary retention following elective joint arthroplasty

**DOI:** 10.1186/s12882-025-04473-w

**Published:** 2025-10-06

**Authors:** Mamdouh Hefny, Raheel Faiz, Alexander Denning, Paul Saunders

**Affiliations:** Orthopaedic Department, South Warwickshire NHS Foundation Trust, Lakin Road, Warwick, Warwickshire, CV34 5BW UK

**Keywords:** Hyponatraemia, Acute kidney injury, Joint replacement, Surgery, Arthroplasty

## Abstract

**Background:**

Acute kidney injury (AKI) and hyponatraemia are common postoperative complications following elective joint replacement surgery, often related to fluid and electrolyte imbalances. Dehydration can contribute to AKI, whereas excessive fluid administration may lead to hyponatraemia. Additionally, urinary retention can mimic AKI by reducing urine output, potentially resulting in unnecessary fluid administration and dilutional hyponatraemia. This study aims to improve postoperative fluid-electrolyte management by differentiating AKI from urinary retention and reducing the incidence of hyponatraemia and fluid overload.

**Methods:**

Data from 616 patients undergoing elective joint replacement surgery over a six-month period were retrospectively reviewed, including electronic records, laboratory results, and fluid balance charts. Following this, an intervention incorporating routine bladder ultrasound scans into fluid management was implemented prospectively. The incidence of AKI and hyponatraemia before and after the intervention were compared using the chi-square test to evaluate the effectiveness of routine bladder scanning.

**Results:**

The incidence of AKI was 1.2%, while hyponatraemia was observed in 38.3% of patients pre-intervention. After introducing routine bladder scanning, the incidence of AKI remained unchanged; however, the incidence of hyponatraemia significantly decreased to 6.5%. Statistical analysis demonstrated a chi-square value of 30.33 (*p* < 0.00001), and, with Yates correction, 28.93 (*p* < 0.00001), indicating a significant reduction in hyponatraemia incidence following the intervention.

**Conclusion:**

Hyponatraemia is a common complication following joint replacement surgery that warrants clinical attention. Implementing routine bladder scanning as part of postoperative fluid management can significantly reduce its prevalence. Ongoing education and targeted interventions are recommended to optimise patient outcomes.

**Clinical trial number:**

Not applicable. We were advised that as no new interventions were carried out there was no need to register this study.

## Introduction

Joint arthroplasty, including total knee replacement (TKR) and total hip replacement (THR), is a highly successful procedure for patients with end-stage joint arthritis. Over 100,000 such surgeries are performed annually in the UK, making them amongst the most common orthopaedic operations. Despite excellent outcomes, these major surgeries carry risks of postoperative complications that can affect recovery. Maintaining fluid and electrolyte balance is crucial for optimal postoperative outcomes.

Acute kidney injury (AKI) and hyponatraemia are among the most frequently encountered postoperative complications following joint arthroplasty [1, 2, 7, 11]. Both conditions are manifestations of fluid-electrolyte imbalance and are associated with significant morbidity and mortality [11].

AKI after joint replacement surgery contributes to adverse outcomes and increased healthcare burden [5, 10, 12]. While some aspects of AKI may be preventable, it is important to recognise that it is a multifactorial condition, not entirely avoidable. In the UK, efforts such as mandatory AKI audits across National Health Service (NHS) hospitals have helped reduce its incidence and improve management [3].

Hyponatraemia, defined as a serum sodium concentration below 135 mmol/L, is another serious postoperative complication with substantial clinical and financial implications [13, 15]. It is classified as mild, moderate, or severe, based on sodium levels, and symptoms can range from nausea and weakness to neurological deficits such as ataxia and cerebral oedema [15]. In surgical patients, hyponatraemia is often dilutional, caused by excess fluid administration, and has been identified as an iatrogenic complication with significant clinical consequences [9, 15]. Despite its frequency, hyponatraemia following orthopaedic surgery has received less attention compared to AKI, and there is limited literature addressing this complication [11].

Urinary retention, a common postoperative issue, can reduce urine output and mimic AKI, potentially leading to inappropriate fluid administration and further exacerbation of hyponatraemia.

Bladder ultrasound is a non-invasive, painless intervention and offers a rapid method to detect urinary retention by showing post-void urinary volume, enabling differentiation from AKI and guiding appropriate fluid management. The use of bladder scanning has been shown to be a useful adjunct in assessing urinary volume post-joint replacement surgery [4].

This interventional study was conducted in a high-volume district general hospital to identify the incidence of AKI and hyponatraemia following elective joint arthroplasty. The study further evaluates the introduction of routine bladder ultrasound scanning as a tool for the early detection of urinary retention, aiming to improve fluid-electrolyte balance and reduce the incidence of hyponatraemia in the perioperative period.

## Methodology

This interventional study was conducted following an initial retrospective observational case series based on multiple local service improvement projects within the Orthopaedics Department at South Warwickshire NHS Foundation Trust.

Two data collection phases were performed at a single high-volume hospital involving patients undergoing elective joint replacement surgery. The retrospective review included 616 patients over a six-month period. Data collected included electronic medical records, blood test results, and fluid balance charts. All surgeries were performed by the same group of surgeons using consistent anaesthetic techniques before and after the intervention involving bladder scanning. Preoperative and postoperative urea, creatinine, and sodium levels were recorded. Patients’ gender, American Society of Anaesthesiologists (ASA) grade (reported as median and interquartile range), age at surgery, perioperative fluid intake, fluid balance, type, and duration of surgery were documented. Patients’ demographics and surgical summaries are shown in Table 1.

### Inclusion criteria

All patients undergoing lower limb arthroplasty were included.

### Exclusion criteria

Trauma patients undergoing joint replacement surgery were excluded.

AKI was diagnosed by the urine component criteria and creatinine-based criteria according to the kidney disease Improving Global Outcomes (KDIGO) staging system in the postoperative period following joint replacement surgery, based on blood tests taken on day one postoperative onwards. Hyponatraemia was defined as serum sodium concentration below 135 mmol/L and further classified as mild (130–135 mmol/L), moderate (125–129 mmol/L), or severe (< 125 mmol/L).

**Table 1:** Patient demographics and surgical summary

Patient Demographics and Surgical Summary	
Category	Value
Total Patients	540
Procedures (First-time candidates)	
- Hip Replacements	239
- Partial Knee Replacements	59
- Total Knee Replacements	242
Patients with metabolic disease (e.g., diabetes)	65%
ASA Grade (median [IQR])	2 [2–3]
Mean Age (years)	71
Sex Ratio (Male: Female)	1:2
Patients on antihypertensives (specifically ACE Inhibitors)	72.60%
Patients on other medications (diuretics, other antihypertensives)	25%
Ethnicity	
- White Caucasian	80%
- Other (Black, Asian, etc.)	20%

All patients had fluid balance charts maintained. Postoperative AKI cases were identified based on creatinine criteria and correlated with fluid balance data. Similarly, patients with postoperative hyponatraemia were documented and analysed in relation to fluid intake and output.

Following the retrospective phase, an intervention was introduced involving routine bladder ultrasound scanning to aid fluid management. This prospective phase included 76 patients, where bladder volume was assessed postoperatively in the recovery room. Urine output and fluid intake were monitored closely. If patients did not void within 4 h of surgery, a second bladder scan was performed, and the volume was compared to the baseline recorded in recovery. If the volume in the urinary bladder increased, fluids were not administered; thus, urinary retention was diagnosed and managed. If the volume in the urinary bladder was not increased compared to the baseline volume on the second bladder scan, AKI was diagnosed with the aid of blood tests and fluids were administered.

The incidence of AKI and hyponatraemia was compared between the retrospective and prospective cohorts. Statistical analysis was conducted using the chi-square test of independence to evaluate the association between bladder scan use and reduction in incidence of hyponatraemia and AKI. A p-value of less than 0.05 was considered statistically significant.

## Results

### Patient cohort and surgical characteristics

The initial data collection included 540 patients undergoing elective lower limb joint replacement over six months. Procedures included 239 hip replacements, 242 total knee replacements, and 59 partial knee replacements (Table 2). The median ASA grade was 2 (IQR 2–3), and the median age was 71 years. The female-to-male ratio was approximately 2:1.

**Table 2:** Incidence of hyponatraemia by procedure type

Procedure Type	Patients with Hyponatraemia, *n* (%)	Mild, *n* (%)	Moderate, *n* (%)	Severe, *n* (%)
Total Cohort (540)	207 (38.3)	164 (79.2)	36 (17.4)	7 (3.4)
Hip Arthroplasty (239)	105 (44)	75 (71)	26 (25)	4 (4)
Total Knee Arthroplasty (242)	90 (37)	76 (84)	12 (13)	2 (3)
Partial Knee Arthroplasty (59)	12 (22)	8 (69)	4 (31)	0 (0)

### Incidence of AKI

The incidence of AKI in this group of patients was 1.2% (7 patients in 540 cohort). All were stage one, diagnosed by the creatinine-based criteria and completely resolved on follow-up during their hospital stay (range 1 to 3 days).

### Incidence and severity of postoperative hyponatraemia

Hyponatraemia (serum sodium < 135 mmol/L) occurred in 207 patients (38.3%). Breakdown by severity was: mild in 164 (79.2%), moderate in 36 (17.4%), and severe in 7 (3.4%) patients (Table 2).

Only patients with severe hyponatraemia required specialist medical intervention, including treatment with hypertonic saline and cardiac monitoring.

### Associations with age, ASA grade, and preoperative sodium

There was a trend towards increasing age and ASA grade with hyponatraemia severity (Table 3). Median ages were 72.6, 77.4, and 79.6 years for the mild, moderate, and severe hyponatraemia groups, respectively. Median ASA grades increased from 2 in the mild group to 3 in the severe group. Patients with low preoperative sodium were more likely to develop postoperative hyponatraemia (54% mild, 32% moderate, 14% severe), compared to patients with normal preoperative sodium (82% mild, 17% moderate, 1% severe).

**Table 3:** Patient characteristics by hyponatremia severity

Patient Characteristics by Hyponatraemia Severity			
**Characteristic**	**Mild**	**Moderate**	**Severe**
Median Age (years)	72.6	77.4	79.6
Median ASA Grade	2	2–3 †	3
Preoperative Sodium: Low (%)	54%	32%	14%
Preoperative Sodium: Normal (%)	82%	17%	1%

**†** ASA grades in the moderate group were distributed between grades 2 and 3

### Fluid intake and timing of surgery

Increased perioperative fluid intake (oral and intravenous) correlated with hyponatraemia severity. Median fluid intake on the day of surgery was 2,449 mL overall, increasing from 2,440 mL (mild) to 2,742 mL (moderate) and 2,911 mL (severe). Patients operated on earlier in the day received more fluids: those operated between 08:00–12:00 received approximately 2,793 mL, compared to 1,965 mL for those after 14:00. Correspondingly, hyponatraemia incidence was higher in patients operated before noon (59%) compared to those operated after 12:00 (38%), consistent with a dilutional effect hypothesis (Table 4).

**Table 4:** Perioperative fluid intake and hyponatraemia by severity and surgery time

Perioperative Fluid Intake and Hyponatraemia by Severity and Surgery Time				
Variable	**Mild**	**Moderate**	**Severe**	**Overall**
Median Fluid Intake on Day of Surgery (mL)	2,440	2,742	2,911	2,449
Time of Surgery	Approx. Fluid Intake (mL)	Hyponatraemia Incidence (%)		
08:00–12:00	2,793	59%		
After 14:00	1,965	38%		

### Impact of bladder scan intervention

After review of these results, a bladder scan protocol was introduced prospectively in 76 patients. Bladder volumes were measured within one-hour post-surgery and repeated after four hours if the patient had not voided. This protocol aimed to differentiate low urine output due to urinary retention or AKI and optimize fluid management to prevent dilutional hyponatraemia.

In the prospective group, 5 patients (6.5%) developed postoperative hyponatraemia, representing a significant reduction compared to the retrospective cohort (38.3%, *p* < 0.00001, chi-square = 30.33, Yates correction = 28.93). The incidence of AKI remained low and unchanged. These findings suggest that bladder scan-guided fluid management effectively reduces hyponatraemia without increasing AKI risk.

## Discussion

The overlapping definitions and presentations of AKI and Postoperative urine retention (POUR) create challenges in managing patients with low urine output after surgery, as these conditions require different treatments. Effective fluid and electrolyte management is central to postoperative care, especially considering the interplay between AKI, urinary retention, and hyponatraemia. Understanding the relationships between these complications can enhance patient management and reduce postoperative morbidity.

The findings suggest that routine postoperative bladder scanning to measure urinary volume could provide valuable baseline data. Comparing initial bladder volumes with subsequent scans (e.g., at four hours post-surgery) helps differentiate between urinary retention and AKI as causes of low urine output. An increase in bladder volume indicates urinary retention, while a stable volume suggests AKI. This distinction may reduce unnecessary fluid administration and lower the risk of dilutional hyponatraemia.

We observed that patients with low postoperative urine output often receive increased oral or intravenous fluids, which correlates with the severity of subsequent hyponatraemia diagnosed on the first postoperative day. This relationship highlights the risk of fluid overload when management decisions rely solely on urine output criteria, which are common to all AKI staging systems.

AKI is a postoperative complication associated with morbidity, mortality, and financial burden [6, 14]. In the UK, heightened awareness, recognition, and auditing have contributed to a declining AKI incidence. Literature reports AKI rates up to 10% following hip or knee arthroplasty [7], but in our series, the incidence remained under 2%, likely reflecting adherence to best practices and active auditing.

Conversely, postoperative hyponatraemia in our study was notably higher at 30%. Although this incidence aligns with some reports, it remains a concerning and underappreciated complication. Increased perioperative fluid administration—often intended to prevent AKI when low urine output is observed—may inadvertently contribute to hyponatraemia.

Misinterpreting postoperative urinary retention as AKI can lead to inappropriate fluid overload, worsening retention [1], and increasing hyponatraemia risk, a serious condition linked to morbidity and mortality [9]. The use of bladder scanning has been shown to be a useful adjunct in assessing urinary volume post-joint replacement surgery [4]. This tool facilitates differentiation between POUR and AKI, thereby reducing unnecessary fluid administration and potentially decreasing hyponatraemia incidence.

Our data also indicate that advanced age and higher ASA scores are associated with increased risk of postoperative hyponatraemia, consistent with findings from Kunze et al. [8], who identified age ≥ 73 and ASA ≥ 2 as key predictors in a large cohort undergoing joint replacement surgery.

Limitations of this study include its retrospective design and lack of a controlled comparator group. However, the benefits observed with the bladder scan intervention rendered randomization ethically challenging. Further prospective studies are warranted to validate these findings and refine the proposed management algorithm. While our work focuses on joint replacement surgery, the principles may be broadly applicable across surgical specialties.

## Conclusion

AKI, urinary retention, and hyponatraemia are among the most frequently encountered postoperative complications following joint replacement surgery. These complications are integrated components of the fluid-electrolyte balance of postoperative patients and are associated with significant morbidity and mortality. The great attention given to the prevention of AKI has considerably reduced its prevalence, and it is yet to observe similar actions taken to manage the increased incidence of hyponatraemia. The routine use of bladder scans may be a helpful tool in avoiding the overlap between establishing a diagnosis of POUR and AKI and, consequently, the prevention of iatrogenic hyponatraemia.

## Data Availability

Data is provided within the manuscript.

## Abbreviations

AKI: Acute Kidney Injury
ASA: American Society of Anaesthesiologists
THR: Total Hip replacement
TKR: Total Knee replacement
POUR: Postoperative urinary retention
